# New frontiers in metabolomics: from measurement to insight

**DOI:** 10.12688/f1000research.11495.1

**Published:** 2017-07-19

**Authors:** Eli Riekeberg, Robert Powers

**Affiliations:** 1 Department of Chemistry, University of Nebraska-Lincoln, Lincoln, NE, USA

**Keywords:** Metabolomics, Metabonomics, Biomarkers, Multiblock Analysis, Dynamic Nuclear Polarization, Chemometrics

## Abstract

Metabolomics is the newest addition to the “omics” disciplines and has shown rapid growth in its application to human health research because of fundamental advancements in measurement and analysis techniques. Metabolomics has unique and proven advantages in systems biology and biomarker discovery. The next generation of analysis techniques promises even richer and more complete analysis capabilities that will enable earlier clinical diagnosis, drug refinement, and personalized medicine. A review of current advancements in methodologies and statistical analysis that are enhancing and improving the performance of metabolomics is presented along with highlights of some recent successful applications.

## Introduction

Metabolomics is a rapidly growing field of study that endeavors to measure the complete set of metabolites (generally considered to be the intermediates and products of cellular metabolism less than 1 kDa in size) within a biological sample (that is, the metabolome) in order to achieve a global view of the state of the system ^1^. Typically, metabolomics is focused only on characterizing the water-soluble metabolites, whereas lipidomics is a specialized discipline that investigates only lipids ^2^. Water-soluble metabolites are part of a mobile, open biological system and as a result can readily interact and communicate with the environment, including the microbiome ^3^. This is also true for some lipids but to a much lesser extent. Consequently, metabolomics has become an essential resource for systems biology because of its unique perspective relative to genomics and proteomics. Numerous studies have measured the relative upregulation or downregulation of genes or proteins to infer changes in biological function. However, it has been shown that even for common metabolic processes, such as glycolysis, a change in the cellular concentration of an enzyme does not necessarily lead to a proportional change in metabolic flux ^4^. Thus, whereas genomics and proteomics identify what *might* happen, metabolomics identifies what *is* actually happening in the system. This realization demands a different perspective and requires the measurement of transcriptional, proteomic, and metabolomic data in order to obtain a complete picture of the system’s response to environmental or genetic stress.

As another illustration, a “silent mutation” does not produce an observable change in phenotype despite an alteration in a gene or protein product. Therefore, metabolomics profiling can be used to decode the function of silent mutations, such as the *Pfk26* and *Pfk27* genes in *Saccharomyces cerevisiae* that both encode the glycolytic/gluconeogenesis regulator phosphofructokinase 2 ^5^. Via co-response and cluster analysis, these genes were observed to exhibit similar metabolite profiles which differed from other genes impacting energy metabolism. For these reasons, methods to directly measure metabolite concentrations within cells, tissues, organs, or other biological samples are crucial for fully understanding a system when traditional omics studies (for example, genomics, proteomics, and transcriptomics) are deemed insufficient. To date, nuclear magnetic resonance (NMR) ^6^ and mass spectrometry (MS) ^7^ have been the primary analytical techniques used to characterize a metabolome. NMR and MS are typically combined with univariate and multivariate statistical methods to identify major metabolite changes and to identify potential biomarkers ^8^. Nevertheless, despite the tremendous growth in the field, critical protocols and techniques are still under development. Herein, we present recent advances in methodologies and statistical analysis that are enhancing and improving the performance of metabolomics while extending the applications in which metabolomics can play a significant role.

## Essential components of a metabolomics study

Conceptually, an untargeted metabolomics study is quite simple. Biological samples are obtained from two or more experimental groups to be compared (healthy versus diseased, wild-type versus gene knockout, and so on) and the metabolites are extracted. These metabolic extracts then are measured by using numerous instrumental techniques, of which NMR and liquid chromatography (LC)-MS are the most common. The resulting spectra then are subjected to statistical analysis techniques such as principal component analysis (PCA) and orthogonal projection onto latent structures (OPLS) to determine the most significant spectral features that define each group ^9, 10^. Finally, these spectral features then can be assigned to distinct metabolites and metabolic pathways by using spectral libraries of known metabolites ^11, 12^. In this manner, untargeted metabolomics is discovery-based since it reveals previously unknown information about how a system responds to environmental or genetic stress. Conversely, targeted metabolomics focuses on analyzing a specific set of metabolites on the basis of some prior knowledge about the system. As a result, targeted metabolomics studies tend to be more sensitive and quantitative and have a higher reproducibility and a lower false-positive rate relative to untargeted metabolomics.

Protocols for obtaining and extracting the metabolome have been well developed and exhaustively reviewed for a wide range of biological samples, including cell cultures, urine, blood/serum, and both animal- and plant-derived tissues ^13– 16^. Although these protocols are readily available, the variable stability of metabolites means that even minor changes in procedure can have a major impact on the observed metabolome. The fast turnover rate of enzymes and the variable temperature and chemical stability of metabolites require that metabolomics samples be collected quickly and handled uniformly and that all enzymatic activity be rapidly quenched in order to minimize biologically irrelevant deviations between samples that may result from the processing protocol ^16, 17^. Thus, the optimization of the sample preparation protocol is essential to a successful metabolomics study. Conversely, the most likely source of bias is improper handling of the metabolomics samples. An important consideration is that the complete metabolome cannot be captured in a single extraction protocol. This is in stark contrast to modern genomics, which can reliably cover the entire genome of an organism. A metabolomics extraction protocol will usually focus on only a subset of metabolites (for example, water-soluble metabolites or lipids). Furthermore, an extraction protocol may focus on either a highly reproducible and quantitative extraction of a restricted set of metabolites (that is, targeted metabolomics) or the global collection of all possible metabolites (that is, untargeted metabolomics) with a possible reduction in precision. In general, 200 to 500 metabolites may be observed by targeted metabolomics, whereas upwards of 1,500 metabolites have been detected in untargeted metabolomics studies ^18^.

Following extraction and subsequent data collection, the final and perhaps most crucial step is metabolite assignment, which typically is accomplished by a comparison with spectral libraries of known metabolites. This is not a trivial task since the number of possible metabolites can be prohibitively large, and a large segment of the metabolome is either unknown or lacking a reference spectrum. For example, the human metabolome is estimated to contain around 150,000 metabolites ^18^, but the Human Metabolome Database ^11^ contains only around 74,000 metabolites (as of 1 June 2017). Thus, there are still many unknown metabolites and a true estimate of the size of the human metabolome is challenging ^19^. Another complicating factor is that different organisms may have completely unique metabolomes. For instance, plants have over 45,000 known secondary metabolites ^20^. Finally, there may be ambiguities in making a metabolite assignment because of chemical shift overlap or identical masses (for example, isomers). As a result, the assignment of metabolites to spectral features may be as low as 4 to 5% ^21^.

## A tale of two methods: mass spectrometry or nuclear magnetic resonance?

Perhaps the most important choice that can be made in a metabolomics study is which instrumental platform is used. Although a wide range of instruments have been used for metabolomics, including capillary electrophoresis, infrared spectroscopy, and Raman spectroscopy, only NMR and MS are routinely used for metabolomics. NMR and MS are often applied in metabolomics investigations because of their inherent complementarity, which results from their distinct advantages and disadvantages ^22^. NMR is highly reproducible and quantitative, has simple sample preparation protocols, and is able to measure analytes over a wide range of solvent conditions ^23^. Despite these advantages, the main limitation of NMR is its low sensitivity, which restricts its application to measuring the most abundant metabolites in the sample (micromolar to millimolar range). This has been noted as a significant hurdle that has slowed the widespread adoption of NMR by the metabolomics community ^22, 24^. Conversely, the high sensitivity and low detection limits of MS enable the detection of subtle metabolic changes that are invisible by NMR. With this increase in sensitivity, the detection of thousands of peaks is relatively common ^25^, but untargeted MS metabolomics studies often are not quantitative in nature. Since MS detectors rely on ionization processes, MS is restricted to detecting metabolites that readily ionize. Correspondingly, a significant reduction in observable metabolites may occur depending on the specifics of the sample being considered ^26^. For a detailed overview of the utility of various MS detectors to metabolomics, see the review article by Dunn *et al*. ^27^ (2005).

MS also suffers from reproducibility problems since contaminants within the sample can change the ionization efficiency of metabolites ^2^. Specifically, quantitation is challenging in untargeted MS since peak intensity is dependent on ionization efficiency, which varies between metabolites and also is strongly dependent on experimental conditions that may result in varying ion suppression ^28^. One issue of particular relevance to MS is the relatively narrow nominal mass and mass defect distribution of the metabolome which results in significant peak overlap ^29^. This can be resolved by coupling MS to a chromatographic method, most commonly LC or gas chromatography (GC), to resolve overlapping peaks and to aid in the metabolite identification based on retention time and the properties of the stationary phase.

GC was the first separation technique applied to the analysis of metabolic mixtures; for example, GC-MS was used to identify biomarkers for diagnosing phenylketonuria in 1970 ^30^. GC-MS is particularly beneficial for the analysis of volatile metabolite mixtures since minimal sample preparation is required; in some cases, samples can be directly analyzed. Furthermore, a number of applications of GC-MS uniquely involve detecting volatile metabolites; two examples are the measurement of exhaled breath condensates for diagnosing lung cancer ^31^ and the monitoring of volatile paper degradation products from historic books ^32^. An obvious disadvantage of GC-MS is its reliance on analyte volatility, where metabolites of low volatility or low temperature stability may be modified or destroyed ^17^. Limited metabolite volatility can be overcome through the use of derivatization schemes, but derivatization is time-consuming. More importantly, differences in the efficiency of the derivatization ^33^ and differences in the stability of the derivatized metabolites ^17^ may dramatically perturb the apparent concentrations of the metabolites, possibly leading to an erroneous biological conclusion.

LC was not widely used for metabolomics until the 1980s ^27^ and this was due to technical limitations with interfacing LC and mass spectrometers. A main advantage of LC over GC is that most metabolites can be detected intact and without modification from a deravitizing agent. Additionally, LC provides an accurate analysis of thermally unstable or reactive metabolites since the separation typically occurs at room temperature. However, the introduction of a liquid phase does introduce a higher variability in retention times ^34^, an increase in ion suppression due to matrix effects ^35^, and a lower resolution relative to GC.

NMR and MS tend to observe a distinct set of metabolites from the same metabolomics sample. Consequently, there is a growing trend in metabolomics to perform tandem studies in which the same sample is analyzed by both NMR and MS ^36– 39^. In this manner, the coverage of the metabolome is significantly increased by taking advantage of the strengths of both methods. NMR identifies trends in metabolic alteration along core metabolic pathways and provides a context for the interpretation of the low-abundance metabolites identified by MS. Of course, the combined use of NMR and MS leads to a proportional increase in data set size with the added complexity of the simultaneous processing, analysis, and interpretation of two dissimilar data types.

### Data processing and interpretation

Metabolomics experiments generate large data sets that require specialized tools for analysis. Numerous software packages for data pre-processing and statistical analysis are available and have been reviewed elsewhere ^40, 41^. Unfortunately, no single software exists that can simultaneously perform all of the critical steps needed for an analysis of a combined NMR and MS data set. Although the statistical techniques applied to NMR and MS data sets are largely the same, each technique requires a unique set of pre-processing tools and algorithms prior to modelling. For example, an NMR spectrum has to be Fourier-transformed and phased, whereas centroiding and de-isotoping are required in MS. Owing largely to these data type–specific processing requirements, newly developed software is almost exclusively restricted to one method or the other. Conversely, there has been minimal effort in developing tools capable of working with both NMR and MS data sets ^40^.

There are two general approaches to integrating NMR and MS data sets into a single coherent study. The first involves samples simply being independently analyzed by each method. The separate data sets then are compared in order to identify consistencies in the metabolic alterations observed by each technique. The main advantage of the approach is simplicity since it does not require any significant protocol changes. Also, the confidence of a metabolite assignment may be significantly increased if it is identified by both methods. Furthermore, a measure of internal consistency may be achieved if metabolite concentrations can be estimated by both methods. However, significant information can be lost during this process since, for example, ambiguity in peak assignments sometimes can be resolved by information from the other method. There is also a lack of statistical correlation since the data sets are independently analyzed. Although the manual curation of independent data sets is the dominant method currently used by metabolomics investigators, it also suffers from reproducibility problems due to potential biases in data interpretation (for example, metabolite assignment methods) among other issues.

The second approach to combining NMR and MS data sets is to simultaneously integrate each data set into a *single* statistical model using a multiblock analysis. Multiblock analysis encompasses a variety of methods that combine multiple data sets prior to conventional multivariate analysis. In addition to combining multiple instrumental data sources ^42^, multiblock analysis has been successfully employed to combine data sets from different omics disciplines ^43^. Multiblock methods are preferable to independent analysis since the relative contributions of each data set still can be quantified, but importantly the larger combined data set is likely to result in models with greater predictive ability and resolving power than either method alone ^44^. However, the software tools to perform multiblock analyses are crude and often rely on custom sets of pre-processing routines using multiple software packages. The lack of integrated analysis tools and software is a major roadblock in metabolomics, especially in light of the growing interest in combining NMR and MS data sets.

## Recent advances in metabolomics

### Dynamic nuclear polarization

NMR metabolomics investigations, especially those concerned with achieving a high confidence in metabolite identification, require two-dimensional NMR methods to resolve the overlap present in one-dimensional spectra. In general, this requires isotopic labelling with NMR-active nuclei like ^13^C and ^15^N because of their low natural abundance. In the last few years, dynamic nuclear polarization (DNP) has evolved from a structural biology tool in the area of solid-state NMR to have potential applications in solution-state metabolomics ^45^. In DNP, a solid, frozen metabolomics sample at about 1.5 K is polarized in the presence of microwave-irradiated free-radicals, which induces a temporary hyperpolarization in spin-active nuclei through a transfer of polarization from electrons to nuclei. The sample then needs to be rapidly melted and transferred to an NMR spectrometer to take advantage of the greatly enhanced sensitivity (>10,000-fold) ^46^. The dramatic increase in sensitivity avoids the need for isotopic labelling, especially for *in vivo* samples, and may permit the detection of low-abundance metabolites. Nevertheless, DNP experiments are limited by T _1_ relaxation rates, resulting in a short measurement window of the dynamically polarized samples. DNP also requires substantial hardware modifications and accessories (for example, microwave generator) to rapidly thaw and shuttle samples back and forth from the NMR spectrometer. DNP has also been applied to ^13^C-labeled metabolites that then are used as a tracer compound for *in vivo* imaging ^47^. This requires close proximity of the polarizer and magnetic resonance imaging spectrometer to allow for rapid transfer, dissolution, and injection of the ^13^C-labeled metabolite given the relatively short T _1_ of 30 to 40 seconds for a ^13^C-labeled carboxyl group. Despite these technical obstacles, DNP has been successfully used to monitor a single metabolite (for example, pyruvate) in living tissue (for example, heart) by magnetic resonance imaging ^48^. Besides the short measurement time, another challenge with the application of DNP to *in vivo* imaging is the limited number of ^13^C-labeled metabolites that can be polarized and tolerated at the concentrations needed for imaging (25 to 80 mM) and that are also a useful biological probe. In addition to pyruvate, bicarbonate, fumarate, urea, glutamine, and dehydroascorbate have been used for *in vivo* imaging ^47^. Despite fundamental issues of reproducibility and limitations in sample preparation, DNP protocols and technology are rapidly advancing and one day could become a routine tool for metabolomics ^49^.

### Disease profiling and personalized medicine

Metabolomics can be used to profile an individual’s responses to a drug treatment or other medical therapy by monitoring metabolite changes in readily obtainable biofluids (for example, blood and urine). A unique advantage of metabolites as biomarkers is the likely occurrence of observing a set of multiple metabolites with distinctly different concentration changes that are correlated with a disease state or treatment response. Correspondingly, multiple metabolites, instead of a single biomarker, are expected to yield a higher sensitivity and selectivity. For example, plasma baseline levels of xanthine, 2-hydroxyvaleric acid, succinic acid, stearic acid, and fructose prior to simvastatin treatment were observed to reliably predict a good or poor response in reducing low-density lipoprotein cholesterol ^50^. The OPLS model yielded a 70% sensitivity and 79% specificity with a corresponding area under the receiver operating characteristic curve of 0.84. Thus, metabolomics can be used to predict whether a patient will respond to a drug in addition to being used as a semi-quantitative prognosis of disease progression.

For example, in a recent study of patients with tuberculosis (TB), urine samples that were collected over the course of a 6-month period became more similar to those of a non-TB control group during the course of first-line anti-TB therapy (for example, isoniazid, ethambutol, or pyrazinamide). Metabolomics has also been successfully employed to identify serum metabolic alterations associated with psoriasis ^51^. Importantly, the metabolomics results were consistent with trends previously observed in genomics and proteomics studies. The metabolome changes were observed to reverse following successful corticosteroid treatment ^52^. Interestingly, the authors identified an increased demand for glutamine, which had not been previously reported in psoriasis ^52, 53^. Glutamine demand is directly associated with diseases characterized by increased cellular proliferation, such as in cancers. A significant alteration in β-isosterol, which is a commonly employed herbal remedy, was also observed. Thus, metabolomics may also be used to identify a patient’s use of alternative treatments outside of his or her physician’s knowledge or recommendation. In this manner, metabolomics may assist in determining whether co-administration of a complementary treatment was beneficial or detrimental to a patient’s therapeutic outcome.

### New trends in data analysis

Much of the data analysis approach in metabolomics has been largely borrowed from the field of chemometrics, which pioneered the application of PCA and PLS to chemical systems ^54^. Although these are powerful statistical tools, the current trend in metabolomics data analysis is evaluating the efficacy of new algorithms and statistical methods to improve group separation and metabolite identification. PCA, PLS, and OPLS are all commonly employed by metabolomics investigators, but newer approaches, including support vector machine (SVM) ^55^, random forest (RF) ^56^, and self-organizing map (SOM) ^57^ algorithms, have all been recently applied to metabolomics data sets.

Despite having been formalized since 1992 ^58^, SVM has been used extensively only in the analysis of gene microarray data, particularly due to its performance on data sets characterized by a large number of variables and few samples ^59, 60^. SVM is also able to identify non-linear relationships that violate the linearity assumptions of PCA and OPLS, making it easily generalizable. SVM has been recently applied in biomarker discovery for ovarian cancer, and a model using serum-derived LC-MS spectra was able to predict disease onset with higher accuracy than the currently accepted method of CA-125 serum monitoring ^61^. A major caveat of SVM is its restriction to binary classification problems: it is able to discriminate between only two experimental groups. Simply, spectra belonging to two experimental groups are represented as points in n-dimensional space, where n corresponds to the number of observed metabolites. A hyperplane then is calculated that best separates the points from the two groups. The coefficients of the calculated hyperplane are used to determine which metabolites are most important for discriminating between the two groups. Although methods have been proposed to extend SVM to multi-class problems, they are often done by breaking down the data set into an ensemble of binary groups that oversimplifies the problem and leads to uninformative models ^62^.

The RF algorithm is a decision tree–based method that uses random subsets of the data to construct multiple models, which then are combined to create an average model in a process known as bootstrap aggregation. In the decision tree method, samples are mapped to a target value (that is, which experimental class the sample belongs to) using a set of variable-based decision rules that separate the samples into groups corresponding to the target value. These newly formed groups can be further subdivided according to new variables, and each “branch” of separation is repeated until the samples can be fully differentiated. The major advantage of the decision tree is its imperviousness to scaling and variable normalization, an extremely common problem in metabolomics data ^63^. The disadvantages include an extreme propensity for overfitting and having extremely poor generalizability that severely limits its utility. RF addresses this limitation by creating an ensemble of partial decision trees that, when combined into an overall model, reduces variance and overfitting ^64^. In particular, the RF algorithm, being relatively unaffected by scaling and normalization and easily handling both large data sets and missing values, is highly adaptable to the realities of real-world data sets. A major disadvantage of RF is that the method requires extensive “tuning” of default parameters by the investigator in order to obtain the best model. Also, the resulting decision tree can be hard to visualize for large data sets ^65^. RFs have shown clinical value: they have been used to determine a set of serum protein and metabolic biomarkers in prostate cancer with higher predictive accuracy than the current prostate-specific antigen biomarker ^66, 67^. See Gromski *et al*. (2015) for an excellent review of the SVM and RF algorithms that also includes comparisons with other mainstream techniques ^65^.

SOM is an approach similar to PCA that reduces multi-dimensional problems to a more easily interpretable low-dimensional grid to visualize natural clustering trends and groupings within a data set. SOM can be applied to the same tasks as PCA but without the biases toward high-variance metabolites. SOMs, like SVM, have the ability to detect non-linear relationships between detected metabolites ^68^. SOMs have been successfully applied to develop biomarkers for early-stage renal cell carcinoma as well as to predict patient response to surgical intervention with a predictive accuracy of 94.74% ^69^. In comparison with the other statistical methods, SOM has been severely limited in metabolomics because of a computationally intensive algorithm and the lack of a pre-packaged software, which has significantly diminished its accessibility to the wider research community ^70^. Nevertheless, the usage of SOMs in metabolomics is steadily rising, and comparative analyses are beginning to demonstrate that SOMs are an acceptable alternative to more traditional clustering algorithms ^71^.

In addition to statistical methods applied directly to spectral profiles, identified metabolites can be used with pathway analysis ^72^ to understand metabolite interactions with known pathways or to discover mechanisms of action in pharmaceutical natural product research ^73^. Metabolomics data sets can generate an overwhelming and seemingly disjointed list of metabolites, which pathway analysis aims to place into a broader biological context by assigning metabolites to relevant metabolic pathways. This is done through a number of software tools that integrate putatively identified metabolites with pathway information from various databases. For example, MetaboAnalyst 3.0 is a suite of metabolomics tools ( http://www.metaboanalyst.ca), which includes modules for metabolite enrichment analysis (MSEA), metabolite pathway analysis (MetPA), and an integrated pathway analysis. The user input is typically a list of metabolites (with or without concentrations) or genes or both. MSEA provides a ranked list of potentially key metabolic pathways based on the observed number of metabolites associated with that pathway (that is, metabolite set enrichment) ^74^. MetPA combines MSEA with a pathway topology analysis to provide an overall pathway analysis to identify the metabolic pathways primarily impacted in the study ^72^. The integrated pathway analysis combines both metabolomics and genomics data with enrichment analysis and topology analysis to again identify the pathways (in rank order) that were primarily impacted in the study ^75^. MetaboSignal ( https://bioconductor.org/) is an alternative approach to pathway analysis which employs directed graphs with network topology approaches to compute centrality measures to correlate gene-metabolite relationships through shortest-path distances ^76^. Thus, unlike the MetaboAnalyst 3.0 tools, the output of MetaboSignal is a network map of gene-metabolite connectivities. Cytoscape ( http://www.cytoscape.org/) is a generalized network interaction and visualization tool that works with a variety of data sets, including metabolomics data. Cytoscape combined with MetScape 3 ( http://metscape.ncibi.org/) ^77^ can generate network maps similar to those of MetaboSignal from metabolomics or genomics data or both ^78^. MetScape uses known pathways from Kyoto Encyclopedia of Genes and Genomes (KEGG) ^79^ and Edinburgh Human Metabolic Network (EHMN) ^80^ databases and gene set enrichment analysis to generate these networks in order to visualize the impacted metabolic pathways. In essence, there is some significant overlap in the capabilities of MetaboAnalyst 3.0, MetaboSignal, and Cytoscape/MetScape 3. Importantly, pathway analysis provides an interaction network that may identify centralized hubs where metabolic pathways coincide or where bottlenecks may occur.

The limited connectivity or altered flow through specific metabolic nodes (that is, change in metabolic flux) may identify functionally essential biological processes ^81^. These essential pathways then can be selectively targeted. By genetically or chemically restricting a potentially essential metabolic pathway, it is possible to ascertain the relevance of the pathway to a systems response to an environmental stress (that is, drug resistance) and potentially reverse or negate the response ^82^.

Pathway analysis also allows for the integration of multi-modal omics data, such as combining gene-expression and metabolomics data to uncover gene and protein functions. For example, metabolite profiles were integrated with genome-wide screening of single-nucleotide polymorphisms (SNPs) to identify the molecular mechanism of the *NAT8* and *PYROXD2* genes. Briefly, SNPs were ranked according to the strength of an association with observed metabolites. The regions where these SNPs occur on the chromosomes then were screened to determine at what position in the genome the gene/protein product responsible for the mediation is stored. With this approach, it was suggested that the *NAT8* and *PYROXD2* genes were responsible for mediation of serum diethylamine levels ^83^, a novel insight for these previously under-annotated genes.

As another illustration, transcriptomic and metabolomic data from *Arabidopsis thaliana* were integrated to characterize the biological response resulting from the over-expression of *PAP1*, a gene known to cause profound accumulation of anthocyanins and to encode a MYB transcription factor regulating flavonoid biosynthesis. The authors were able to correlate the biosynthesis of cyanidin and quercetin derivatives with a specific set of upregulated genes that enabled them to identify the function of two uncharacterized proteins: a flavonoid 3-O-glucosyltransferase and anthocyanin 5-O-glucosyltransferase ^84^. Numerous tools are now available for pathway analysis of metabolomics data ^79, 85, 86^, which will significantly improve data interpretation and simplify our understanding of biological relevance. Thus, pathway analysis is becoming a routine component of a detailed metabolomics analysis.

## Conclusions: What does the future hold?

The recent advancements in metabolomics outlined herein have been shown to enhance its utility in systems biology research and to have a beneficial impact on medical research and personalized medicine. The measurement of metabolomics profiles has been shown to be useful for monitoring treatment efficacies from both pharmaceutical and surgical interventions. As our understanding of the relationship between disease state and the chemical profile of biofluids grows, metabolomics is expected to become a routine approach for monitoring disease development and progression, as a tool for disease diagnosis, and for understanding the underlying molecular mechanisms of drug resistance. Metabolite profiles could be obtained at regular intervals and screened for changes over a patient’s lifetime as a diagnostic tool and a means to monitor a patient’s overall health status. Some of this work has already begun; an ongoing Alphabet (parent company of Google) “moon-shot project” is a baseline study attempting to determine the inherent level of variability in human medical data that is not associated with a disease. Though still in its infancy, a similar approach using metabolomic profiles may be used to determine the inherent variability in biofluid profiles for healthy individuals. In this manner, metabolic profiles associated with disease onset and progression can be easily distinguished from the known variance in healthy individuals.

Some of the biggest challenges remaining in the field of metabolomics involve fundamental limits in experimental methodology. Metabolomics requires relatively high-cost instrumentation and complex data analysis and still suffers from issues of sample-to-sample variability. Although great strides in each of these areas have been made, there is still more work to be done before metabolomics can become a key and routine part of a clinical practice. Nevertheless, metabolomics continues to make important contributions to both medical research and general systems biology studies. In fact, the ability to directly measure metabolite concentration changes by using a targeted NMR or MS approach would greatly benefit investigations into a range of research areas that often are overlooked by other methods. In this manner, a metabolomics assay that targets a select and specific set of metabolites can be used to develop a highly reproducible and quantifiable assay that can be translated into a validated clinical assay.

## Abbreviations

DNP, dynamic nuclear polarization; GC, gas chromatography; LC, liquid chromatography; MetPA, metabolite pathway analysis; MS, mass spectrometry; MSEA, metabolite enrichment analysis; NMR, nuclear magnetic resonance; OPLS, orthogonal projection onto latent structures; PCA, principal component analysis; PLS, projection onto latent structures; RF, random forest; SNP, single-nucleotide polymorphism; SOM, self-organizing map; SVM, support vector machine; TB, tuberculosis.
